# The Effect of Iron Content on the Ammonia Selective Catalytic Reduction Reaction (NH_3_-SCR) Catalytic Performance of FeO_x_/SAPO-34

**DOI:** 10.3390/ijerph192214749

**Published:** 2022-11-10

**Authors:** Zhaoyang Li, Geng Chen, Zhenghua Shao, Haonan Zhang, Xiujuan Guo

**Affiliations:** 1 Faculty of Maritime and Transportation, Ningbo University, Ningbo 315211, China; 2 School of Civil and Transportation Engineering, Ningbo University of Technology, Ningbo 315211, China

**Keywords:** selective catalytic reduction, SAPO-34 molecular sieve, iron, nitrogen oxides

## Abstract

Iron-based catalysts are regarded as promising candidates for the ammonia selective catalytic reduction reaction (NH_3_-SCR) which show good catalytic activity at medium and high temperatures, whereas SAPO-34 molecular sieves have a micro-pore structure and are ideal catalyst carriers. In this paper, four FeO_x_/SAPO-34 molecular sieve catalysts with different iron contents (Fe = 1%, 2%, 3%, 4%) were prepared using an impregnation method. The effect of iron content on the surface properties and catalytic activity was investigated by a series of characterization techniques including XRD, SEM, BET, XPS, H_2_-TPR and NH_3_-TPD. Iron species in the FeO_x_/SAPO-34 catalysts exist in the form of isolated iron ions or well-dispersed small crystals and iron oxide species clusters. With the addition of iron content, the integrity of CHA (chabazite) zeolite structure remained, but the crystallinity was affected. The FeO_x_/SAPO-34 catalyst with 3% Fe loading showed a relatively flat surface with no large-diameter particles and strong oxidation-reduction ability. Meanwhile, more acidic sites are exposed, which accelerated the process of catalytic reaction. Thus, the FeO_x_/SAPO-34 catalyst with 3% Fe showed the best NO conversion performance among the four catalysts prepared and maintained more than 90% NO conversion efficiency in a wide temperature range from 310 °C to 450 °C.

## 1. Introduction

Among environmental pollutants, nitrogen oxides (NO_x_) from industrial and vehicle exhaust gas emissions cause a series of environmental issues including acid rain, photochemical smog, exhaustion of the ozone layer, fine particulate air pollution and harm to human health [1]. The ammonia selective catalytic reduction reaction (NH_3_-SCR) can effectively remove NO_x_; the key technology is the selection of the catalyst [2]. Currently, the best commercially available catalysts for SCR systems are V_2_O_5_–WO_3_/TiO_2_ catalysts, in which the active component, vanadium, is poisonous and has negative effects on the environment. Compared with the commercial V_2_O_5_–WO_3_/TiO_2_ catalysts, the zeolite catalysts are characterized by a higher NH_3_ storage capacity, a higher activity in NH_3_ and NO oxidation reactions, and a higher activity in the standard SCR reaction at low temperatures [3]. In recent years, materials based on transition-metal ion-exchanged zeolites have received significant attention due to the improved NO_x_ reduction performance in a wide temperature range [4]. Iron-based molecular sieve catalysts have higher NO_x_ conversion efficiency in a wide temperature window and less environmental damage [5]. Among them, iron-based molecular sieve catalysts have received wide attention.

Fe-based zeolite catalysts show outstanding SCR activity in a wide temperature range. Ellmers et al. investigated the relationship between Fe content and normalized reaction rates, using Fe/ZSM-5 molecular sieve catalysts prepared using an ion exchange method. In standard SCR, normalized reaction rates reached a maximum at around 0.5 wt% Fe content (Fe/Al = 0.07), which suggests the existence of isolated Fe^3+^ sites and Fe^3+^ in small oligomeric structures. Fast SCR proceeds in the absence of Fe, but reaction rates are poor [6]. Andonova et al. concluded that for the hydrothermal synthesis method with the addition of small amounts of Fe in the Fe/SAPO-34 catalyst, the framework zeolite structure in the parent SAPO-34 sample was slightly affected and with the increase of Fe content; the crystallinity of the sample changes significantly and the size of the unit cell expanded. The lowest Fe loading (0.27 wt%) of the Fe/SAPO-34 catalyst gave the best SCR catalytic activity [7]. Based on the above studies, it was demonstrated that the catalytic activity was closely related to Fe content. In a recent work, the Fe/SSZ-13 catalyst for NH_3_-SCR was successfully prepared using a one-step synthesis method under hydrothermal conditions and showed good NH_3_-SCR catalytic performance. The monomer [Fe-(OH)_2_]^+^ and dimer [HO-Fe-O-Fe-OH]^2+^ are the iron species with relative activity for the Fe/SSZ-13 catalyst [8]. Brandenberger et al. prepared a Fe-ZSM-5 catalyst using a liquid ion exchange method to maintain more than 80% NO conversion efficiency in a wide temperature range (300 to 600 °C). Monomeric iron sites primarily contributed to the SCR activity at temperatures below 300 °C, and the contribution of dimeric iron species became important at temperatures above 300 °C [9]. Gao et al. analyzed the nature of iron sites in the Fe/SSZ-13 catalysts prepared using the aqueous solution ion exchange method, and suggested that the extra framework Fe^3+^ ions, poorly crystallized Fe_2_O_3_ particles and isolated Fe^2+^ ions were the moieties containing Fe species. Isolated Fe^3+^ ions were the active sites for the low-temperature standard SCR reaction, the dimeric sites were the active centers for NO oxidation and NH_3_ oxidation was catalyzed by sites with higher nuclearity [10]. The studies elucidated that the iron species affected the SCR catalytic activity of Fe-based zeolite catalysts. Lai et al. found that the Fe-ZSM-5 catalysts prepared using an ion exchange method in an air atmosphere were beneficial to improving the ability of Fe ionic exchange; however, Fe_x_O_y_ nanoparticles and small oligomeric Fe_x_O_y_ clusters were considerably increased. Isolated Fe^3+^ sites with high SCR activity at low temperatures were dominant in the Fe-ZSM-5 catalysts prepared in an N_2_ atmosphere. The Fe-ZSM-5 catalysts in an N_2_ atmosphere had more Brønsted acid sites and a higher adsorption capacity for NH_3_ species and nitrate species, which was conducive to SCR activity [11]. Shi et al. studied the effect of different solvents on the performance of NH_3_-SCR in the Fe-ZSM-5 catalysts prepared using an impregnation method. When water was used as the solvent, more iron clusters were formed on the zeolite support resulting in poor activity at low temperatures. With methanol as the solvent, surface-adsorbed oxygen and a large amount of Fe^3+^ in the Fe-ZSM-5 catalysts produced good performance at low temperatures. The poor performance at high temperatures is due to excess high-valence iron ions in the Fe-ZSM-five catalysts [12]. Niu et al. considered that the NO conversion of the Fe/SSZ-13 catalyst obtained at a calcination temperature of 500 °C was nearly 100% in the temperature range from 300 to 400 °C, whereas the NO conversion of the catalyst obtained at a calcination temperature of 550 °C did not exceed 50% in the temperature range from 100 to 550 °C. Higher calcination temperature resulted in the formation of more iron species migrating and coalescing, which is unfavorable for the reaction [8]. As shown above, the activity of SCR catalysts was influenced by different preparation conditions.

In a summary of the literature, different Fe content and preparation conditions affect SCR catalytic performance. There are few studies on SAPO-34 molecular sieves, which are characterized by low-cost and excellent catalytic activity. The impregnation method is a common method to prepare supported catalysts and provides a fast and convenient method for zeolite catalyst preparation when compared with the hydrothermal synthesis method [7]. By applying the impregnation method, the irreversible hydrolysis of the SAPO-34 molecular sieve during the liquid phase ion exchange process also can be avoided [6].

In this paper, FeO_x_/SAPO-34 molecular sieve catalysts were prepared using an impregnation method with water as the solvent in an air atmosphere. This study investigated the effects of iron contents on the structure, specific surface area, reduction, acidity and NH_3_-SCR performance of the FeO_x_/SAPO-34 catalysts. The relationship between sample microstructure and SCR catalytic activity was investigated using XRD, SEM, BET, XPS, H_2_-TPR and NH_3_-TPD characterization techniques, and the reasons for the improved catalytic performance of SCR catalysts were analyzed.

## 2. Experimental

### 2.1. Catalyst Preparation

The FeO_x_/SAPO-34 molecular sieve catalysts were prepared using a commercial SAPO-34 molecular sieve by the impregnation method with water as the solvent in an air atmosphere and was performed by mixing 3 g of SAPO-34 molecular sieve powder with the required amounts of Fe(NO_3_)_2_·9H_2_O solution at ambient temperature. Under ultrasonic radiation, it was thoroughly stirred for 2 h and then impregnated for 24 h. The prepared catalysts were dried at 110 °C for 12 h, and subsequently calcined at 600 °C for 5 h. After calcination, the FeO_x_/SAPO-34 molecular sieve catalyst was obtained. To betterinvestigate the relationship between the catalytic activity and iron content, the catalysts were labeled as FeO_x_/SAPO-34-1, FeO_x_/SAPO-34-2, FeO_x_/SAPO-34-3 and FeO_x_/SAPO-34-4 with the Fe loading at 1, 2, 3 and 4 wt%, respectively.

### 2.2. Catalyst Characterization

The phase compositions of all the catalysts were identified using powder X-ray diffraction (XRD). The X-ray tube was operated at 40 kV and 200 mA using Cu-Kα radiation (λ = 0.15418 nm). The X-ray powder diffractograms were scanned at a 5°/min rate in the range from 5° to 90°.

Scanning electron microscopy (SEM, JEOL Ltd., JSM-7800F) was used to observe the morphology and structural features of the catalysts.

Nitrogen adsorption–desorption measurements were performed using an ASAP 2020M physical adsorption analyzer. The samples were degassed for 1 h at 100 °C, and then degassed for more than 3 h at 350 °C; the specific surface areas were obtained using the Brunauer–Emmett–Teller (BET) model. Pore volume and average pore width were evaluated using the Barrett–Joyner–Halenda method.

X-ray photoelectron spectra (XPS) were observed on Thermo ESCAlab250 equipment and the spectra were collected using Al-Kα radiation. The surface charging impact in the measurement process was adjusted by the 285.0 eV value of the C 1 s binding energy.

The H_2_ temperature-programmed reduction experiments (H_2_-TPR) analyze the redox properties of catalyst samples. First, 50 mg samples were pretreated for 0.5 h at 300 °C with a flow of 27 mL min^−1^ nitrogen. Afterward, the samples were cooled to ambient temperature in nitrogen. The samples were heated during the measuring process at a ramping rate of 10 °C min^−1^ to 900 °C.

Before the NH_3_-temperature programmed desorption experiments (NH_3_-TPD), 100 mg of samples were pretreated for 0.5 h at 300 °C in flowing He (30 mL min^−1^). NH_3_ (10 mL min^−1^) was adsorbed for 0.5 h in a He flow (30 mL min^−1^) atmosphere at a temperature of 80 °C. After NH_3_ adsorption saturation, the NH_3_ valve was closed and the system purged with He (30 mL min^−1^) until the curve was stable. To record NH_3_-TPD curves, the samples were heated to 900 °C at a rate of 10 °C min^−1^.

### 2.3. NH_3_-SCR Activity Evaluation

The catalytic activity tests were performed in a fixed-bed reactor system (i.d. = 6 mm) with 2 mL of powdered catalysts (40 to 60 mesh). The simulated exhaust gas contained 400 ppm NO, 400 ppm NH_3_, 6% O_2_ and N_2_ for balance. The total volume flow rate was 1 L/min corresponding to the gaseous hourly space velocity (GHSV) of 30,000 h^−1^. The catalytic activities were measured at 180 to 600 °C. The concentration of the gas stream at the reactor inlet was monitored continuously using a mass flowmeter and the outlet gases were measured using a gas analyzer. Furthermore, the NH_3_-SCR reaction was carried out steadily for 20 min and then data were collected. The NO_x_ conversion efficiency was calculated using the following equation:

$${\mathrm{NO}}_{x}\mathrm{conversion}(\%)=\frac{\phi {\left({\mathrm{NO}}_{x}\right)}_{in}-\phi {\left({\mathrm{NO}}_{x}\right)}_{out}}{\phi {\left({\mathrm{NO}}_{x}\right)}_{in}}\times 100\%,$$

where the subscripts *out* and *in* indicate the NO_x_ concentration at the outlet and inlet, respectively.

## 3. Results and Discussion

### 3.1. Catalyst Characterization Results

#### 3.1.1. XRD

**Figure 1:**
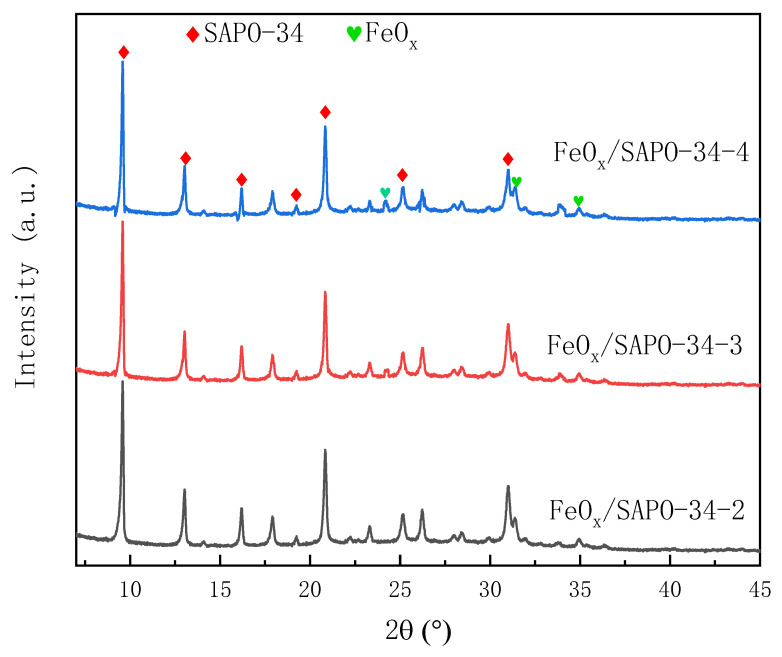
XRD patterns of the samples: FeO_x_/SAPO-34-2, FeO_x_/SAPO-34-3 and FeO_x_/SAPO-34-4.

The XRD patterns of all samples are shown in Figure 1. Determination of the physical phase of catalyst samples was performed using the XRD diffraction angular position and intensity [13,14]. The characteristic diffraction peaks for the FeO_x_/SAPO-34 molecular sieve appear at 2θ = 9.61°, 13.15°, 16.28°, 19.3°, 20.81°, 26.2° and 31° from the XRD patterns, which is consistent with the typical characteristic diffraction peaks of the SAPO-34 molecular sieve. The characteristic peaks for FeO_x_ (2θ = 24.1°, 33.2°, 35.6°, 39.2° and 40.8°) are also present in the spectra and their intensity strengthens with iron loading increase. Meanwhile, the characteristic peak intensity for FeO_x_ strengthens with the weakening of the diffraction peak intensity of SAPO-34. Their crystallinity shows a slightly decreasing trend due to the iron loading increase. This shows that one part of the iron species in the SAPO exists incorporated as isolated ions or well-dispersed small crystals and the other part aggregates on the material surface in the form of iron oxide. This result shows that the main form of the iron species in the samples is isolated ions or well-dispersed small crystals. The isolated ions act as the reaction active sites and are favorable to the catalytic activity of the sample. The small part of the iron species aggregates on the material surface in the form of iron oxide; the agglomerates block the channels of the molecular sieves, which are not conducive to the reaction.

#### 3.1.2. SEM

**Figure 2:**
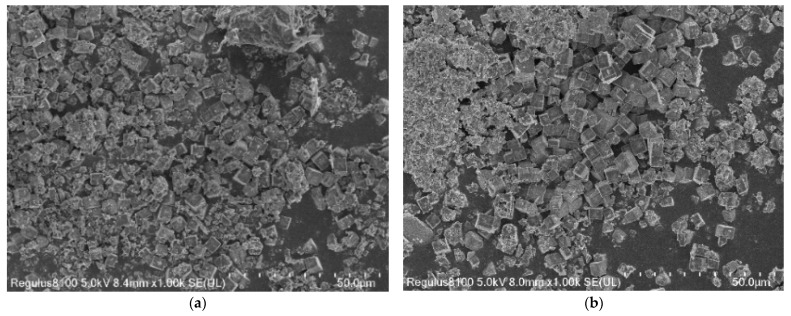

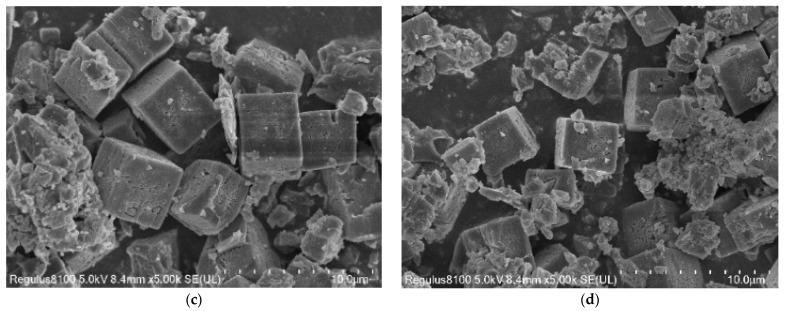
SEM results of the FeO_x_/SAPO-34 samples: (**a**) FeO_x_/SAPO-34-1; (**b**) FeO_x_/SAPO-34-2; (**c**) FeO_x_/SAPO-34-3; (**d**) FeO_x_/SAPO-34-4.

As confirmed by the SEM investigation in Figure 2, all the samples are cubic crystals with crystal sizes of 2 to 10 μm, which is the typical CHA structure. The grain morphology and size of crystals as displayed in Figure 2 suggest that the iron loading increase has an impact on the structural integrity of the catalysts, which is in line with the XRD results. With increasing the amount of Fe in the catalysts, many iron oxide particles are present and agglomerate together on the surface of the block over FeO_x_/SAPO-34; the distribution is heterogeneous, and the grain size is about 50 to 200 nm. Figure 2c, FeO_x_/SAPO-34-3, shows that although some amorphous particles appear on the surface of the samples, the crystal structure of CHA zeolite is not destroyed by Fe doping and the active substance is well dispersed on the surface of the molecular sieve support, which results in the relatively better NH_3_-SCR performance of FeO_x_/SAPO-34-3 molecular sieve catalysts.

#### 3.1.3. BET

**Figure 3:**
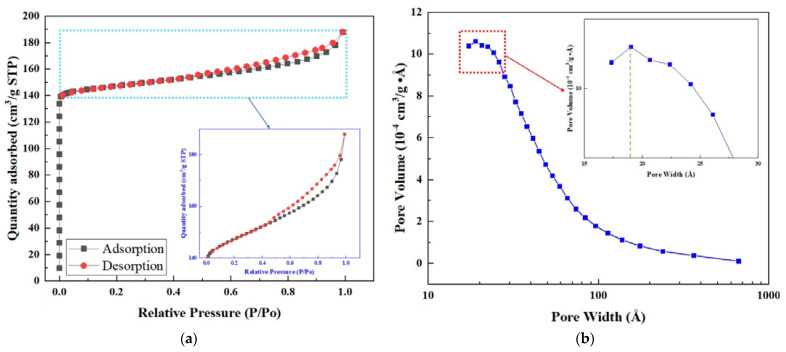
The FeO_x_/SAPO-34-3 molecular sieve sample: (**a**) N_2_ adsorption–desorption isotherms; (**b**) Pore size distribution.

The physical and morphological properties of the SAPO-34 and FeO_x_/SAPO-34-3 are listed in Table 1. The results show that compared with pure SAPO-34, FeO_x_/SAPO-34-3 has a significant decrease in BET surface area and an increase in average pore width but less change in total pore volume. As compared with the SAPO-34, the BET surface area of FeO_x_/SAPO-34-3 decreases from 541.78 cm^2^/g (SAPO-34) to 474.78 cm^2^/g (FeO_x_/SAPO-34), total pore volume decreases from 0.301 cm^3^/g (SAPO-34) to 0.292 cm^3^/g (FeO_x_/SAPO-34), and average pore width increases from 2.01 nm (SAPO-34) to 2.44 nm (FeO_x_/SAPO-34). Combined with the above XRD results, iron species increase in particle size and cover the external surface of FeO_x_/SAPO-34 and partially block the channels, which could result from the fact that the pore structure of the SAPO-34 molecular sieve is reduced, and the BET surface area and pore width is also reduced accordingly. Based on the conclusion above, the BET surface area and pore width of the samples decrease with the excessive increase of iron content. Figure 3a exhibits the N_2_ adsorption–desorption isotherms of the FeO_x_/SAPO-34-3 catalysts; the curves are the I-type N_2_ adsorption–desorption isotherms.

#### 3.1.4. XPS

**Figure 4:**
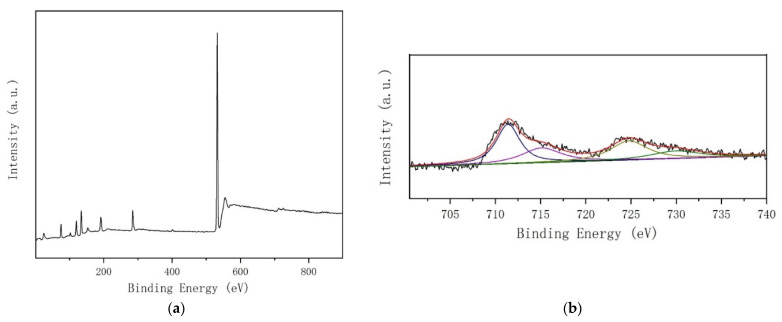
FeO_x_/SAPO-34-3 molecular sieve sample prepared by an impregnation method: (**a**) Full XPS spectrum, (**b**) Fe 2p spectrum.

XPS measurements were performed to study the surface components as well as the chemical state of the elements [15,16] over the FeO_x_/SAPO-34-3 samples. Figure 4a shows the characteristic peaks of Al, Si, P, O and Fe. As shown in Figure 4b, the XPS results of Fe 2p for the FeO_x_/SAPO-34-3 catalyst can be observed. The satellite peak positions of Fe 2p_1/2_ and Fe 2p_3/2_ depend on the ionic state of Fe [17], and are also very sensitive to the oxidation state, whereas the ionic state of Fe can be determined qualitatively by these peaks. Figure 4b indicates that the peak of Fe 2p_1/2_, located at 724.78 eV, is ascribed to Fe^2+^ species and the Fe 2p_3/2_ peak at 711.40 eV, is attributed to Fe^3+^ species. The role of different iron species on the SCR reaction were reported in the literature, and results show that the isolated Fe^3+^ sites were considered as the reaction active site [9]. The Fe contents of the FeO_x_/SAPO-34-3 molecular sieve sample is 0.93 wt%. The areas of the fitting peaks lead to Fe^3+^/(Fe^3+^ + Fe^2+^) = 56.74%.

#### 3.1.5. H_2_-TPR

**Figure 5:**
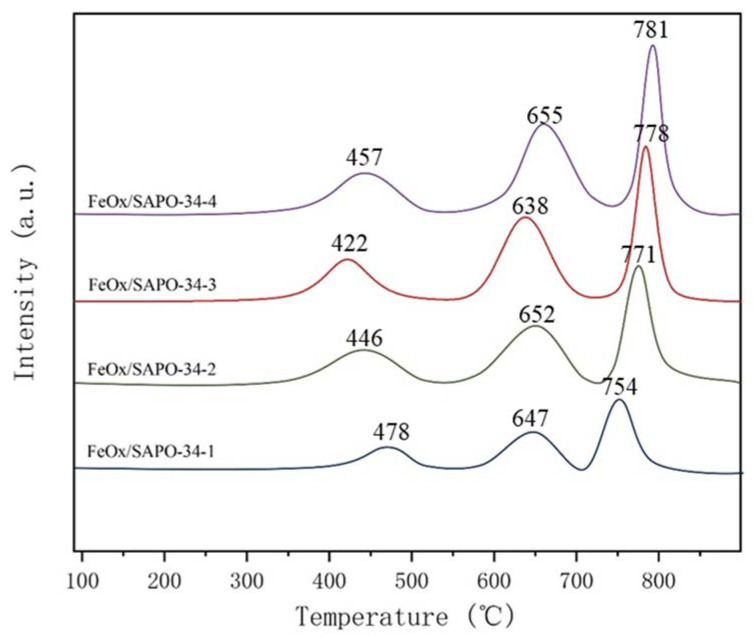
H_2_-TPR spectrum of the FeO_x_/SAPO-34(x) molecular sieve samples prepared by the impregnation method.

H_2_-TPR measurements were carried out to probe the reducibility of species [18,19] and the results are shown in Figure 5. The H_2_ consumption peaks observed from 100 to 900 °C can be divided into three reduction peaks. The reduction peaks in the low-temperature interval are attributed to the reduction of Fe_2_O_3_ to Fe_3_O_4_, the reduction peaks in the medium-temperature interval are attributed to the reduction of Fe_3_O_4_ to FeO, and the reduction peaks in the high-temperature interval are attributed to the reduction of FeO to Fe. The amounts of iron species on the FeO_x_/SAPO-34-3 sample are estimated by reduction peaks in H_2_-TPR profiles. The content of Fe_2_O_3_, Fe_3_O_4_ and FeO are 0.03231 mmol/g, 0.05092 mmol/g and 0.05734 mmol/g respectively. Fe^3+^ is reduced to Fe^2+^ and the low-valence Fe^2+^ is oxidized to Fe^3+^, which involves the re-dox cycle reaction. The FeOx/SAPO-34-3 catalyst shows the largest reduction peak area, indicating the strongest interaction between Fe species and SAPO-34 with the highest content of active Fe species. With the increase of Fe loading, the temperature of the low-temperature reduction peak decreases and then increases, and the reduction temperatures of the four samples in the low-temperature region from low to high are FeO_x_/SAPO-34-3 < FeO_x_/SAPO-34-2 < FeO_x_/SAPO-34-4 < FeO_x_/SAPO-34-1, with the temperatures of 422, 446, 457 and 478 °C, which indicate that the FeO_x_/SAPO-34-3 catalyst has the highest reduction ability and the best low-temperature SCR activity. Finally, the zeolite-based catalysts followed the “E-R” mechanism or “L-H” mechanism in the SCR reaction [14], and the research on the catalytic mechanism of the zeolite-based catalysts was regarded as the follow-up research.

#### 3.1.6. NH_3_-TPD

**Figure 6:**
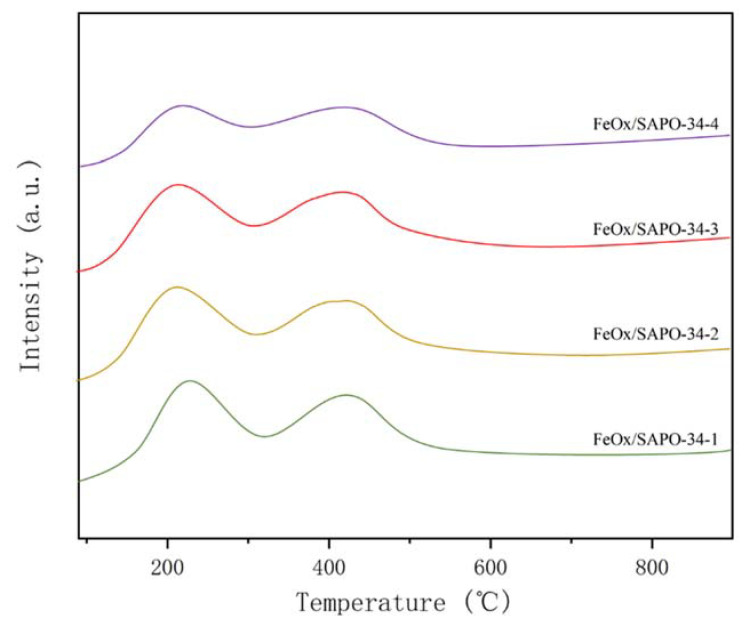
NH_3_-TPD spectrum of the FeO_x_/SAPO-34(x) molecular sieve samples prepared by the impregnation method.

The catalyst surface acidity plays an important role in the NH_3_-SCR reaction [20]. Figure 6 shows the NH_3_-TPD profiles of the FeO_x_/SAPO-34 catalysts in the temperature range of 100 to 800 °C. The relative positions of the ammonia desorption peaks of NH_3_-TPD curves with respect to temperature can provide information on the strength of acid sites over all catalysts [21,22]. As shown in Figure 6, ammonia desorbs in two main peaks near 210 °C and 410 °C respectively. The peak observed at 170 to 240 °C is attributed to the physically adsorbed NH_3_ or ammonium species desorbing at the weak acid sites; the peak at 380 to 430 °C is the NH_3_ desorbed at the strong acid sites. Table 2 illustrates the variation of acid amount for different samples. With the increase of iron loading, some of the acidic sites are consumed resulting in a slow decrease in acidity. The acidic sites and intensity of the catalyst surface affect the adsorption and activation of the reactant NH_3_, but it is not the only decisive factor; therefore, the catalyst NH_3_-SCR activity should be analyzed in combination with other physicochemical properties.

### 3.2. De-NO_x_ Performance of the SCR Catalysts

**Figure 7:**
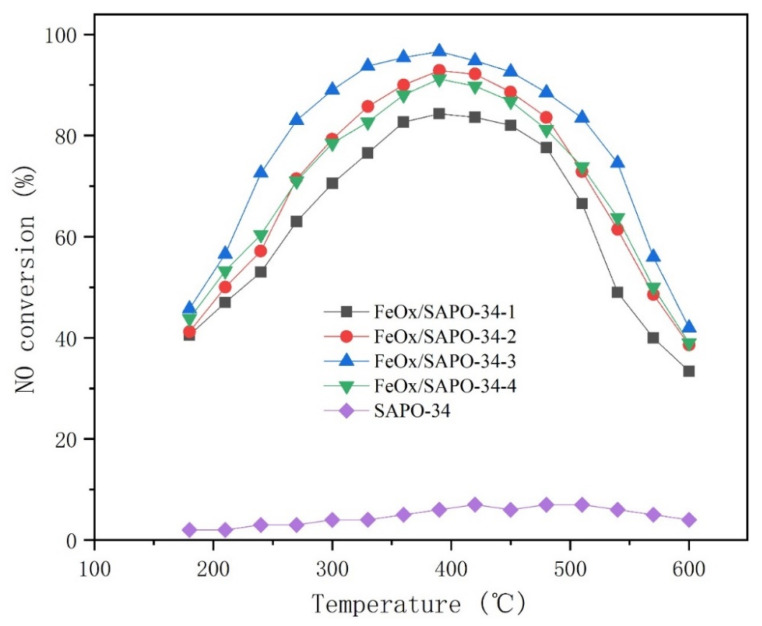
NO conversion of the different samples. Reaction conditions: 400 ppm NO, 400 ppm NH_3_, 6% O_2_ and N_2_ as balance, GHSV 30,000 h^−1^.

The NO conversion efficiency of the FeO_x_/SAPO-34 molecular sieve catalysts is presented in Figure 7. The catalytic activity tests were repeated for all catalysts to check catalyst stability. All data were averaged from the measurements to ensure data reliability. The test temperatures are from 180 to 600 °C. the NO conversion efficiency curves of the samples all show a peak shape, high temperature for initiation of activity and NO conversion efficiency decreases rapidly at high temperature. The NO conversion efficiency temperature window for >90% is narrow and has an optimum SCR activity at 380 to 400 °C. The SCR catalytic performance of the obtained samples is consistent with that reported in the literature under similar conditions [6,7]. The FeO_x_/SAPO-34-3 molecular sieve catalyst has the best SCR catalytic performance, maintaining more than 80% NO conversion efficiency in the temperature range of 260 to 530 °C, more than 90% NO conversion efficiency in the temperature range of 310 to 450 °C and reaching the highest NO conversion efficiency of 97.87% at about 380 °C. The SCR catalytic performances of FeO_x_/SAPO-34-2 and FeO_x_/SAPO-34-4 are relatively close, and the SCR catalytic activity of FeO_x_/SAPO-34-2 is slightly higher than that of FeO_x_/SAPO-34-4 at 270 to 490 °C. The FeO_x_/SAPO-34-4 is slightly better in the rest of the temperature range. The FeO_x_/SAPO-34-2 maintains more than 80% NO conversion efficiency in the temperature range of 310 to 490 °C, more than 90% NO conversion efficiency in the temperature range of 360 to 440 °C and achieves the highest NO conversion efficiency of 92% at about 390 °C. The FeO_x_/SAPO-34-1 SCR shows the worst catalytic activity, maintaining more than 80% NO conversion efficiency in the temperature range from 350 to 460 °C, with the highest NO conversion efficiency of only 84.3% at 390 °C. Overall, with the increase in temperature, the SCR catalytic activity of the molecular sieve catalysts first increases and then decreases.

As shown in Figure 7, the NO conversion efficiency of all samples shows a down-trend in the high temperature range. According to the literature [8,9], the oligomeric iron species were not only the active sites for the SCR reaction in the high temperature range but were also the active sites for nonselective oxidation of NH_3_. NH_3_ nonselective oxidation caused insufficient reductant required for the NO reduction reaction and reduced the SCR reaction activity. With the increase of Fe content, the catalytic activity of the FeO_x_/SAPO-34 molecular sieve catalyst first increases and then decreases. The appropriate amounts of active species covering the surface of the SAPO-34 molecular sieve can maintain good dispersion and have less influence on the pore structure of the molecular sieve; therefore, a good NO conversion efficiency can be obtained. Fe species in FeO_x_/SAPO-34-4 have exceeded the maximum carrying capacity of the molecular sieve surface and have appeared to agglomerate into larger particles and block the pore structure of the molecular sieve and the NO conversion efficiency began to decrease.

## 4. Conclusions

A series of FeO_x_/SAPO-34 molecular sieve catalysts with different Fe loadings (1%, 2%, 3%, 4%) were prepared using an impregnation method, among which the molecular sieve with 3% Fe loading has the best SCR catalytic activity with NO conversion efficiency greater than 90% in the temperature interval of 310 to 450 °C and reaches the highest NO conversion of 96% at about 380 °C. The introduction of Fe has some effects on the structure and performance of the SAPO-34 molecular sieve. The pure SAPO-34 molecular sieve provides a large specific surface area and, after loading iron by impregnation, the Fe active substance is dispersed on the surface of the molecular sieve as well as within the pore channels, resulting in a significant decrease in specific surface area. XRD analysis shows that the characteristic peaks of CHA-type molecular sieves change little, and the characteristic adsorption peaks of the active substance iron oxide can be observed, which indicate that the active substance does not destroy the intact structure of the SAPO-34 molecular sieve, and the active component forms larger grains. The SEM shows that with the increase of Fe loading, the surface of the molecular sieve will become rougher, and the Fe species will cover the surface of the material or exist in the interstices of the pore structure, resulting in the reduction of the specific surface area and pore volume of the material. Compared with other samples, the surface of the FeO_x_/SAPO-34-3 molecular sieve is relatively flat, and no large-diameter particles appear. Compared with other samples, the low- and medium-temperature reduction peak temperatures of the FeO_x_/SAPO-34-3 molecular sieves are shifted to the low-temperature region, which has stronger redox ability and accelerates the oxidation process of NO. Meanwhile, more acidic sites are exposed, which improved the adsorption and activation of NH_3_ on the catalyst surface.

## Figures and Tables

**Table 1:** Specific surface areas, pore volume and average pore diameter for all the catalysts.

Sample	Micro Pore		Full Pore		
	Specific Surface Area (m^2^/g)	Pore Volume (cm^3^/g)	Specific Surface Area (m^2^/g)	Pore Volume (cm^3^/g)	Average Pore Diameter (nm)
SAPO-34	534.4	0.29	541.8	0.30	2.0
FeO_x_/SAPO-34-3	415.5	0.20	474.8	0.29	2.4

**Table 2:** Calculation of acid quantity for the FeO_x_/SAPO-34 molecular sieve samples.

Sample	Low-Temperature Peak Ammonia Desorption Volume (mmol/g)	High-Temperature Peak Ammonia Desorption Volume (mmol/g)	Total Ammonia Desorption (mmol/g)
FeO_x_/SAPO-34-1	0.79	0.52	1.31
FeO_x_/SAPO-34-2	0.77	0.50	1.27
FeO_x_/SAPO-34-3	0.72	0.46	1.18
FeO_x_/SAPO-34-4	0.65	0.41	1.06

## Data Availability

Not applicable.
